# Sensor-integrated hip and knee prostheses: advances, challenges, and future perspectives

**DOI:** 10.3389/fbioe.2025.1721499

**Published:** 2026-02-02

**Authors:** Xiang-Dong Wu, Zhixiong Zhao, Da Lu, Hongyi Shao, Dejin Yang, Yixin Zhou

**Affiliations:** 1 Department of Orthopaedic Surgery, Beijing Jishuitan Hospital, Capital Medical University, Fourth Clinical College of Peking University, National Center for Orthopaedics, Beijing, China; 2 Beijing Research Institute of Traumatology and Orthopaedics, Beijing Jishuitan Hospital, Capital Medical University, Fourth Clinical College of Peking University, National Center for Orthopaedics, Beijing, China; 3 Beijing Advanced Innovation Center for Biomedical Engineering, School of Biological Science and Medical Engineering, Beihang University, Beijing, China

**Keywords:** digital phenotypes, orthopedic phenotyping, remote monitoring, sensor-integrated hip and knee prostheses, smart implant, total hip arthroplasty (THA), total knee arthroplasty (TKA), wireless telemetry

## Abstract

Total joint arthroplasty consistently alleviates pain and improves function in patients with end-stage joint disease. Nevertheless, up to 10% of hip and 20% of knee recipients remain dissatisfied after surgery, and registry data indicate that approximately one in five implants requires revision within 25 years, most commonly due to aseptic loosening, mechanical instability, or periprosthetic joint infection. Conventional postoperative surveillance relies on intermittent clinic visits and imaging, leaving a critical blind spot in our understanding of implant performance during daily activities. To address this gap, research has turned to fully implantable smart prostheses, such as hip and knee implants, embedded with sensors and low-power wireless telemetry that enable real-time monitoring of *in vivo* conditions. This review traces the evolution from early instrumented prototypes to the first commercially available smart knee; outlines enabling technologies, including sensing, communication, powering, and system integration; and summarizes clinical applications and early human data across this development continuum. Smart implants capture objective *in vivo* parameters that are not accessible to routine follow-up, including joint loads, range of motion, spatiotemporal gait metrics, and temperature, thereby enabling orthopedic phenotyping through dynamic, longitudinal digital representations of recovery trajectories and complication patterns. Fully implantable smart prostheses have the potential to shift arthroplasty toward continuous remote monitoring and proactive, precision follow-up care. Coupled with robust clinical decision-support systems and rigorous long-term evaluation, these technologies may usher in a new era of intelligent joint arthroplasty, with the potential to improve outcomes and extend implant longevity.

## 1 Introduction

Total hip arthroplasty (THA) and total knee arthroplasty (TKA) are among the most successful surgical procedures in modern medicine and consistently alleviate pain and improve mobility for millions of patients with end-stage hip or knee arthritis (Bhandari et al., 2019). Procedure volumes continue to increase worldwide; in the United States alone, approximately 1.25 million total joint arthroplasty (TJA) procedures are performed annually, including hip and knee arthroplasties (Agency for Healthcare Research and Quality, 2025). Projections indicate that the volume of THA is expected to increase by approximately 176% by 2040 and 659% by 2060, whereas the volume of TKA procedures is expected to increase by approximately 139% by 2040 and 469% by 2060 (Shichman et al., 2023). Despite the overall success of these interventions, a substantial subset of patients report suboptimal outcomes, with approximately 10%–20% remaining dissatisfied after TKA and 5%–10% dissatisfied after THA (Khatib et al., 2020; Okafor and Chen, 2019). Additionally, as the annual volume of primary THA and TKA continues to increase, the expanding number of hip and knee prostheses *in situ* means that even modest long-term attrition translates into a substantial absolute revision burden; long-term registry data indicate that by 25 years, approximately 40% of artificial hips and 18% of artificial knees have been revised (Evans et al., 2019a; Evans et al., 2019b).

Failure mechanisms of THA and TKA are multifactorial. Common modes can be consolidated into four domains (Nolan and Phillips, 1996; Pitta et al., 2018; Kelmer et al., 2021; Tarazi et al., 2021):

- Mechanical/kinematic—including instability or dislocation, malalignment or component malrotation, patellofemoral maltracking/overstuffing, impingement, and arthrofibrosis;
- Interface/materials—wear-related osteolysis with aseptic loosening, corrosion/trunnionosis (THA), and liner fracture/dissociation;
- Biological—periprosthetic joint infection; and
- Structural—periprosthetic fracture.

Once implanted, an artificial joint functions as a load-bearing “organ” that interacts continuously with complex biomechanical and biochemical environments over decades. However, a critical challenge in contemporary practice is the lack of continuous, real-world monitoring following surgery. Current routine surveillance relies on infrequent clinical assessments and periodic imaging, providing only static, episodic snapshots of postoperative status. Key parameters cannot be effectively monitored in real time, including joint contact forces, *in vivo* loading patterns, kinematic conflicts (defined as implant geometry-guided motion conflicting with native soft-tissue-guided knee motion), micromotion at the bone–implant interface, component alignment, and local physiological signals such as temperature, pH, or biochemical indicators of incipient infections (Vickers et al., 2021; Wang et al., 2024). Consequently, early pathological processes often progress silently during daily activities and remain unrecognized until symptomatic or structural deterioration occurs; for example, polyethylene wear can generate debris that drives osteolysis long before radiographic loosening is evident, and low-grade infection or subtle instability is likewise frequently missed until substantial damage has developed, at which point complex, invasive, and costly revision is commonly required (Nolan and Phillips, 1996; Lee et al., 2023; Burny et al., 2000).

This surveillance limitation creates a clear clinical need for continuous, real-world monitoring of implant health. In response, fully implantable, sensor-integrated “smart” hip and knee prostheses have been developed to deliver continuous or on-demand *in vivo* data for rehabilitation via wireless telemetry, to support early detection of complications, and to enable more individualized rehabilitation (Ledet et al., 2018; Iyengar et al., 2021). These systems quantify objective signals in daily life across three broad domains:

- Biomechanical loading (axial and mediolateral joint loads and peak forces);
- Kinematics and activity (range-of-motion trajectories, activity levels, and spatiotemporal gait parameters such as cadence and step-time asymmetry), and
- Local physiologic surrogates (temperature, with emerging surrogates including pH and biochemical markers).

The resulting continuous data streams can be distilled into dynamic, longitudinal digital phenotypes of joint function and peri-implant biology, an approach we refer to as orthopedic phenotyping. By mapping device-derived signals onto clinically interpretable constructs, this framework may facilitate earlier detection of complications, targeted interventions, and personalized rehabilitation (Ledet et al., 2018; Iyengar et al., 2021).

In recent years, rapid advances in microelectronics, low-power wireless telemetry, and biointegrated sensors have moved smart arthroplasty devices from concept toward clinical practice. Over the past three decades, multiple sensor-integrated designs for THA and TKA have been developed (Taylor et al., 1998; Morris et al., 2001; D'Lima et al., 2005; D'Lima et al., 2006; D'Lima et al., 2007; Heinlein et al., 2007; Heinlein et al., 2009; Mündermann et al., 2008; Meswania et al., 2008; Crescini et al., 2009; Crescini et al., 2011; Damm et al., 2010; Bergmann et al., 2012; Sauer et al., 2013; Silva et al., 2013; Cachão et al., 2019; Safaei et al., 2020; Wijayaratna et al., 2021; Soares Dos Santos et al., 2021; Peres et al., 2022; Cushner et al., 2021; Gordon et al., 2025), and in 2021, the first FDA-cleared sensor-enabled knee (Persona IQ®) entered clinical practice (Cushner et al., 2021; Gordon et al., 2025; Chang et al., 2025), establishing an archetype for connected orthopedic implants integrated with secure digital health platforms that link patients and clinicians. Rather than replacing standard assessments, these fully implantable smart prostheses augment postoperative care by continuously quantifying the implant’s biomechanical and physiological state *in vivo* and enabling timely, data-driven interventions, which are difficult to achieve with episodic clinic visits alone (Sauer et al., 2013; Cachão et al., 2019; Soares Dos Santos et al., 2021; Anil et al., 2022).

Building on this conceptual shift, the central hypothesis of this review is that sensor-integrated hip and knee prostheses can transform arthroplasty follow-up by enabling orthopedic phenotyping and continuous, remote, objective monitoring. Accordingly, we provide a comprehensive overview of current research and developments in smart joint implants, highlighting the enabling technologies, clinical applications, challenges, and considerations for broader implementation. We first trace the historical evolution of smart prosthetic designs from early instrumented prototypes to recently commercialized devices. We then detail the sensor technologies, wireless communication, powering strategies, system integration and packaging, and biocompatibility and safety considerations for implantable electronics. Additionally, we summarize clinical evidence on patient outcomes and highlight representative international studies that illuminate *in vivo* joint mechanics and recovery trajectories, providing a global perspective on this emerging field. Finally, we discuss the multifaceted challenges, including technical, biological, regulatory, and ethical issues that must be addressed for routine adoption, and we conclude with future directions, milestones required to demonstrate clinical benefit, and anticipated advances.

## 2 Evolution of smart joint prostheses

**FIGURE 1:**
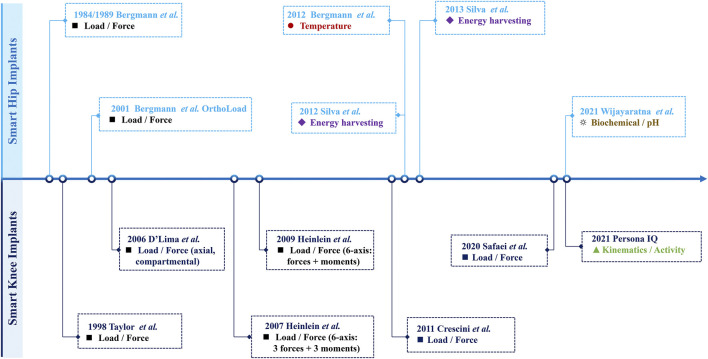
Chronological evolution of sensor-integrated smart joint prostheses.

Efforts to instrument orthopedic implants for *in vivo* measurement in TJA date back several decades. As early as 1966, Rydell and colleagues attempted to quantify *in vivo* joint loads and kinematics using instrumented implants (Rydell, 1966), but contemporary technology, particularly limited battery capacity and telemetry, imposed severe constraints (Davy et al., 1988; Graichen et al., 1988). Early results revealed substantial variability and limited accuracy, reflecting the nascent state of sensor and telemetry capabilities at the time (Bergmann et al., 1984). Nonetheless, these studies established a proof-of-concept that instrumented implants could measure *in vivo* intra-articular forces and kinematics during functional activities (Graichen and Bergmann, 1991; Graichen et al., 1999). A significant milestone followed in 1998, when Taylor et al. reported the first patient-derived telemetric data from a knee implant (Taylor et al., 1998). They instrumented a hinged distal femoral prosthesis used for reconstruction after tumor resection with strain gauges and an inductive telemetry link, enabling real-time transmission of joint force data during walking (Taylor et al., 1998). Although this was a single-patient study involving a customized prosthesis, it demonstrated the feasibility of fully implantable wireless joint-force monitoring and laid critical groundwork for more generalizable smart-implant designs (Meswania et al., 2008). However, these early systems were bespoke, research-grade platforms that prioritized proof-of-concept biomechanical measurements over scalable, long-term clinical deployment (Figure 1).

### 2.1 Smart knee implants

**FIGURE 2:**
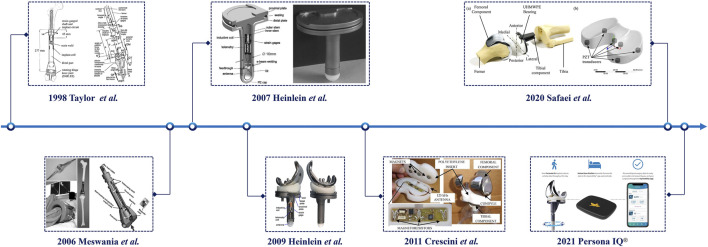
Representative architectures of smart knee implants.

In the early 2000s, the development of the first generation of sensor-integrated prostheses for TKA accelerated. Notably, D’Lima and colleagues at the Scripps Clinic engineered an instrumented tibial tray in 2004–2005 (D'Lima et al., 2005; D'Lima et al., 2006; D'Lima et al., 2007). This custom tibial tray incorporated an internal cavity that housed four uniaxial load sensors, one in each quadrant and separated by support columns. Implanted during standard TKA, these sensors measured axial forces across the tibial plateau, enabling real-time calculation of the total joint load and medial–lateral load distribution. The initial prototype, which was successfully implanted and tested in a single patient, provided the first *in vivo* tibial force data from a smart knee implant (D'Lima et al., 2006). However, this early design focused on maximizing axial compression and could not resolve shear or rotational components (D'Lima et al., 2006), prompting iterative improvements in subsequent systems (D'Lima et al., 2007). As such, it exemplified a research-oriented design that maximized axial load–measurement fidelity in a very small cohort rather than a device optimized for long-term, large-scale clinical deployment. By the late 2000s, more sophisticated multi-axis telemetric implants had emerged. Heinlein et al. introduced a six-degree-of-freedom instrumented tibial component featuring an array of 12 strain gauges embedded in the tibial stem, enabling simultaneous reconstruction of three orthogonal force components and the corresponding three moments about the implant axes (Heinlein et al., 2007). Implanted and tested *in vivo*, the system generated comprehensive datasets characterizing knee joint loading patterns during typical daily activities (Heinlein et al., 2009). In a cohort of five patients, the peak tibiofemoral contact forces during routine activities reached approximately 3–4 times body weight, and the load distribution across compartments varied according to the activities of daily living (Kutzner et al., 2010). This high-fidelity, six-degree-of-freedom (DOF) construct further extended the research lineage of instrumented knees but at the cost of increased mechanical complexity, labor-intensive calibration procedures, and a form factor that is difficult to translate into routine clinical use (Figure 2).

These groundbreaking studies provided unprecedented insight into *in vivo* joint contact forces and moments experienced by knee implants during walking, stair ascent/descent, and other activities of daily living. The resulting data have since been utilized to refine implant design and optimize surgical techniques (Halder et al., 2012; Schwiesau et al., 2014; Grupp et al., 2017). In particular, insights into compartmental load distribution have informed strategies for component alignment and soft-tissue balancing, whereas precise measurements of peak joint contact forces have guided postoperative activity recommendations (Damm et al., 2017; Kutzner et al., 2017). At the same time, these datasets were derived from small, highly selected cohorts, and the strain-gauge–based telemetry systems remain susceptible to calibration drift and long-term signal instability *in vivo*, underscoring the need for robust drift-compensation schemes and long-term validation in larger, more diverse patient populations.

### 2.2 Smart hip implants

Parallel efforts advanced the development of smart hip implants, with foundational contributions from Bergmann et al. In 1984, Bergmann and colleagues quantified hip joint loads using animal models, providing initial biomechanical insights that informed subsequent human investigations (Bergmann et al., 1984). In 1989, Bergmann et al. reported *in vivo* hip joint contact stresses during physiotherapy in humans (Bergmann et al., 1989).

**FIGURE 3:**
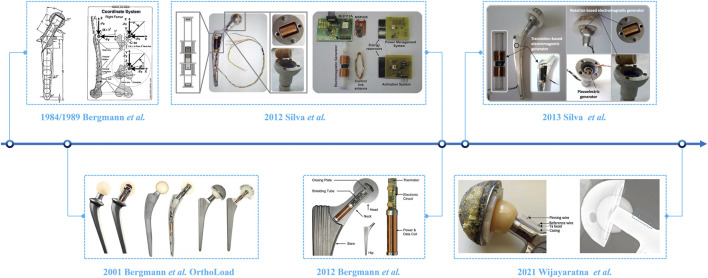
Representative architectures of smart hip implants.

In 1993, using telemetric total hip prostheses, they further measured hip joint forces in two patients during walking and running, demonstrating substantial variability in hip joint loads across different activities (Bergmann et al., 1993). In 2001, Bergmann et al. investigated frictional heating in hip implants during daily activities (e.g., walking) and reported that implant temperature varied with body weight and activity type, emphasizing potential risks to periarticular tissues and the rationale for low-friction bearing materials (Bergmann et al., 2001). In 2012, they introduced a sensor-equipped hip prosthesis capable of directly measuring hip joint contact forces while simultaneously monitoring temperature at the implant interface (Bergmann et al., 2012). This telemetric femoral implant incorporates strain sensors to capture joint loads, along with a thermistor to detect local temperature fluctuations (Bergmann et al., 2012). Temperature monitoring was intended to identify adverse reactions such as excessive friction or early-stage infections, both of which may present as sensor-detectable local temperature increases (Bergmann et al., 2012). Data from this advanced, multisensor prosthesis were transmitted via wireless telemetry, exemplifying the sophisticated integration of multimodal sensing in a load-bearing orthopedic implant (Bergmann et al., 2012). However, similar to the instrumented knees, these telemetric hips primarily served as high-fidelity research platforms in very small patient cohorts, and their long-term performance is constrained by issues such as encapsulation durability and potential drift of strain- and temperature-sensing elements *in vivo*, which complicate the interpretation of longitudinal measurements and highlight the need for robust drift-compensation strategies and long-term validation (Figure 3).

Concurrent with these developments, other research groups explored novel diagnostic sensing modalities and power solutions. In 2013, Sauer et al. developed a prototype hip implant that employed accelerometer-based vibration analysis to detect aseptic loosening. With an accelerometer embedded in the femoral component, subtle changes in the vibration profile at the implant–bone interface could be monitored, indicating potential aseptic loosening, a leading cause of implant failure (Sauer et al., 2013). This concept enabled the implant to detect aseptic loosening, thereby addressing one of the most common causes of implant failure. Researchers also recognized the challenge of powering smart implants and began investigating energy-harvesting solutions. Silva et al. proposed a hip prosthesis incorporating four miniature energy harvesters, comprising electromagnetic and piezoelectric generators, to convert kinetic energy from joint motion and vibration into electrical power (Silva et al., 2013). Although primarily theoretical at the time, and with realistic harvested power expected to be in the microwatt range for typical activities, this work highlighted strategies for self-powered or, more plausibly, hybrid smart implants that supplement implanted batteries by leveraging the mechanical energy available during limb movement, thereby reducing reliance on primary cells (Silva et al., 2013; Singh et al., 2025). By the 2020s, research into sensor-integrated implants had expanded to include biochemical monitoring. Wijayaratna et al. developed a pH-sensitive hip implant featuring a polyacrylic acid-based hydrogel, which contracts under the acidic conditions characteristic of septic joint infections (Wijayaratna et al., 2021). This contraction was measurable radiographically via an embedded radiopaque marker, allowing detection of potential infections well before overt clinical symptoms. However, this approach remains at a preclinical stage, and pH shifts are not specific to infection, highlighting both the promise and the limitations of biochemical sensing in this context. Together, these developments broaden the diagnostic capabilities of smart prostheses beyond purely mechanical sensing, while underscoring the need for more specific biochemical markers and long-term *in vivo* validation.

The historical development of experimental smart knee and hip implants is shown in Figure 1, highlighting their diverse sensing capabilities, including load measurement, vibration-based detection of loosening, energy-harvesting modules, and pH sensing for infection surveillance. Despite their innovative design, most early smart implants remained largely experimental for many years. Validation was typically confined to laboratory settings or small patient cohorts, and widespread clinical adoption did not occur. The first-generation devices provided narrow data streams, often limited to a single modality (e.g., axial load), and faced several technical constraints. For example, embedding sensors within load-bearing components such as tibial trays could perturb native load-transfer pathways, potentially biasing measurements. Sensor resolution and durability were also constrained by the technology available at the time, and the form factors of sensors and electronic components limited feasible placement within the implant construct. Moreover, many of these designs did not undergo extensive mechanical endurance testing (e.g., fatigue, wear, and hermetic sealing) or comprehensive biomechanical validation prior to clinical use. Additionally, their reliance on strain-gauge–based load sensing with long lead wires and analog telemetry renders them vulnerable to calibration drift *in vivo*, driven by biofouling, encapsulant creep, and material fatigue, thereby complicating the longitudinal interpretation of the recorded signals. Consequently, their long-term clinical performance and overall safety remain uncertain, and these platforms are not yet suitable for large-scale, routine implantation.

In summary, these studies collectively established the technical feasibility of smart orthopedic implants. Investigators have instrumented knee and hip prostheses with strain gauges, pressure sensors, accelerometers, and even chemical sensors, using wireless telemetry to stream *in vivo* data from inside the body. While these pioneering studies provided crucial insights and laid the groundwork for subsequent generations, they also underscored critical limitations, including a narrow sensing scope, bulky hardware, and unproven long-term reliability. Rapid advances in microelectronics and sensor technology over the past decade have begun to mitigate these constraints, enabling more compact, energy-efficient architectures and paving the way for clinically viable devices.

This technological evolution reflects a distinct divergence in engineering philosophies. The early experimental telemetric hips and knees exemplify a research-grade lineage that maximized measurement fidelity and biomechanical insight at the expense of device complexity, power consumption, and scalability. In contrast, newer commercially deployed systems, such as the Persona IQ® smart knee, prioritize low-power, lower-bandwidth kinematic sensing that can be sustained for years, is integrated with cloud-based analytics, and can be deployed at scale in standard implant geometries. Highlighting this shift from high-fidelity research tools to clinically deployable monitoring devices is essential to understanding how smart joint prostheses have evolved and why future designs must balance scientific detail against practicality, regulatory constraints, and long-term reliability, as summarized in Table 1.

**TABLE 1:** Comparative synthesis of research-grade and commercial smart joint prostheses systems.

Feature	Research-grade systems (e.g., Bergmann telemetric hip; D’Lima/Heinlein instrumented knees)	Commercial system (e.g., Persona IQ® smart knee)
Primary goal	High-fidelity biomechanical validation (forces/moments; *in vivo* mechanics)	Remote monitoring and recovery tracking (functional/kinematic phenotyping)
Implant form factor	Research/custom implants (THA + TKA; limited runs)	TKA with sensorized tibial extension (standardized, commercially available)
Sensing modality	Strain gauges (loads/forces ± moments; incl. 6-DOF reconstruction), thermistors	Inertial sensing–derived gait and ROM metrics (e.g., step count, walking speed)
Data characteristics	High-bandwidth raw waveforms; session-based acquisition (tens–hundreds Hz)	Longitudinal summary metrics; periodic background synchronization (home hub → cloud)
Power strategy	Inductive coupling (often battery-free; external coil required)	Primary battery (reported service life up to ∼10 years)
Communication	Inductive/near-field telemetry (short range; real-time when coupled)	Low-power RF/Bluetooth LE-class link to home base station; cloud integration
User Burden	High (coil alignment/lab sessions; external equipment)	Low (passive monitoring with home base station)
Evidence Base (typical)	Small, highly selected cohorts (n = 1–10); primarily biomechanical endpoints	Clinical deployment observational cohorts (often n > 100); clinical workflow integration
Clinical status/Regulatory	Research-only/investigational use	FDA De Novo granted; commercially available
Key advantages	Direct *in vivo* force/moment quantification; gold-standard datasets for validation	Scalable long-term monitoring; minimal patient burden; enables remote follow-up
Key Limitations	Calibration drift; reliance on external coupling; limited scalability	Lower biomechanical fidelity (no force data); finite battery life

Abbreviations: Total hip arthroplasty (THA); total knee arthroplasty (TKA); degrees of freedom (DOF); range of motion (ROM); hertz (Hz); radio frequency (RF); Low Energy (LE); sample size (n); U.S. Food and Drug Administration (FDA).

## 3 Sensing, communication, and powering in implantable smart prostheses

Building a fully implantable smart joint prosthesis requires the multidisciplinary integration of sensing, communication, and power-supply technologies within a standard orthopedic implant. This section outlines sensor hardware and on-implant integration, data communication pathways, power supply and energy management strategies. Also, it addresses critical considerations such as biocompatibility, hermetic packaging and feedthroughs, electromagnetic compatibility (including MRI), and overall device longevity.

### 3.1 Sensor hardware and integration

Smart implants incorporate various sensor types (Table 2) to monitor the mechanical and biological state of the joint. In this section, we structure the discussion by sensor type and, for each class, summarize the working principle, measured parameters, typical applications in hip and knee prostheses, interfacing for data transmission, output signal characteristics, and implications for power consumption and system-level energy management.

**TABLE 2:** Overview of sensor modalities, performance specifications, and clinical readiness in smart joint prostheses.

Sensor category	Primary parameters monitored	Representative applications	Clinical status/TRL (approx.)	Commercial component examples	Key implant-relevant specs/Integration notes
Strain-/Load-based (strain gauges/Load cells)	Joint contact forces; compartmental loads; center of pressure	Instrumented tibial trays (e.g., D’Lima) and telemetric hip stems (e.g., Bergmann)	Research/Human Pilot (TRL 6–7)	Vishay; Kyowa; HBM (foil gauges)	Gauge factor ∼2.0; fatigue life >10^7^ cycles; analog bridge output (requires excitation + ADC); sampling 50–200 Hz; Power: Tens–hundreds µW
Multi-axis 6-DOF (force/Moment)	3D forces + 3D moments (Fx, Fy, Fz; Mx, My, Mz)	6-DOF instrumented tibial components (e.g., Heinlein)	Research/Human Pilot (TRL 6–7)	ATI; Kistler (refers to sensor tech used in test rigs)	Range: up to 50 kN; Bandwidth: >10 kHz (captures impacts); accuracy: ∼0.1% FS; highest info density but high complexity
Inertial sensors (IMUs: Accel + Gyro)	Orientation; ROM; step count; gait speed; activity phenotypes	Persona IQ® smart knee (commercial); Activity monitoring studies	Commercial (TRL 9)	Bosch (BMI series); STMicroelectronics (LIS/LSM)	Range: ±16 g/±2000 dps; resolution: 12–16 bit; Low power (10–500 µA); digital output (I2C/SPI); standard in wearables
Temperature sensors (thermistors)	Interface temperature; frictional heating; inflammation signals	Telemetric hip implants (friction monitoring)	Research (TRL 5–7)	TE connectivity; Amphenol; TDK	Accuracy: ±0.1 °C; drift: <0.5 °C/year; sampling: 0.1–1 Hz; minimal power; key for detecting infection/friction
Vibration/Piezoelectric	Vibration signatures (loosening); dynamic strain; micromotion	Aseptic loosening detection prototypes; energy harvesting	Preclinical/Lab (TRL 3–5)	TE connectivity (Piezo film); Murata	Bandwidth: 100 Hz–10 kHz (micromotion signatures); high impedance; passive potential (LC tags) or self-powering
Biochemical/pH	pH (infection); inflammatory markers (IL-6); wear debris	pH-responsive hydrogels for early infection detection	Preclinical (TRL 2–3)	ISFET platforms (predominantly research prototypes)	Sensitivity ∼55 mV/pH; Nernstian response; challenges: Biofouling, drift, and need for stable wet interface

Abbreviations: Technology Readiness Level (TRL); Gauge factor (GF); Analog-to-digital converter (ADC); Hertz (Hz); Microwatt (µW); Degrees of freedom (DOF); Three-dimensional (3D); Force components along x/y/z-axes (Fx, Fy, Fz); Moment/torque components about x/y/z-axes (Mx, My, Mz); Kilonewton (kN); Kilohertz (kHz); Full Scale (FS); Inertial measurement unit(s) (IMU/IMUs); Range of motion (ROM); Accelerometer (Accel); Gyroscope (Gyro); Degrees per second (dps); Microampere (µA); Inter-Integrated Circuit (I2C); Serial Peripheral Interface (SPI); Degree Celsius (°C); Electromagnetic (EM); Inductor–capacitor (LC); Potential of hydrogen (pH); Interleukin-6 (IL-6); Ion-sensitive field-effect transistor (ISFET); Millivolt (mV).

#### 3.1.1 Strain- and load-based sensors

Working principle and measured parameters: Strain- and load-based sensors exploit the relationship between mechanical deformation and electrical signal. Metal-foil or semiconductor strain gauges bonded to load-bearing regions of femoral or tibial components experience slight changes in resistance proportional to local strain, which can be calibrated against known loads to reconstruct joint contact forces and bending moments (D'Lima et al., 2013; D'Lima et al., 2008). In instrumented knees and hips, these sensors typically measure axial compression, mediolateral shear, and flexion/extension moments over a range spanning body-weight–equivalent loads (≈1–5× body-weight) during activities of daily living (Iyengar et al., 2021; Funk and Crandall, 2006).

Applications and function in smart hip and knee prostheses: Early research implants (e.g., D’Lima’s instrumented tibial tray and Heinlein’s 6-DOF tibial component) embedded strain-gauge bridges in stems or trays to quantify compartmental and global tibiofemoral loads, generating detailed *in vivo* load profiles during walking, stair negotiation, and sit-to-stand tasks (D'Lima et al., 2005; D'Lima et al., 2006; D'Lima et al., 2007; Heinlein et al., 2007). Telemetric hip prostheses developed by Bergmann and colleagues similarly used strain gauges in the femoral stem to reconstruct three-dimensional hip contact forces (Bergmann et al., 2012; Bergmann et al., 1984; Graichen and Bergmann, 1991; Graichen et al., 1999; Kutzner et al., 2010; Halder et al., 2012; Schwiesau et al., 2014; Grupp et al., 2017; Damm et al., 2017; Kutzner et al., 2017; Bergmann et al., 1989; Bergmann et al., 1993; Bergmann et al., 2001). These measurements have informed implant design, component alignment strategies, and soft-tissue balancing, and they continue to serve as the gold standard for validating computational models and wear simulators.

Output signal, interfacing, and data transmission: Strain gauges and load cells produce small differential resistance changes, typically arranged in Wheatstone-bridge or multibridge configurations. On-implant electronics excite these bridges with a constant voltage or current, amplify the resulting millivolt-level signals using low-noise instrumentation amplifiers, and digitize them via analog-to-digital converters (ADCs) before transmission. Data are then telemetered using inductive links, proprietary RF telemetry, or low-power radiofrequency modules, as discussed in the communication section.

Power requirements and system-level implications: Bridge excitation currents must be carefully limited to avoid self-heating and excessive power draw. Depending on the number of bridges, the sampling frequency (often 50–200 Hz for gait-related applications), and the duty cycle, strain-gauge subsystems typically consume on the order of tens to hundreds of microwatts, making them a dominant contributor to the overall power budget in high-fidelity, load-sensing implants. Consequently, designers must trade off measurement bandwidth and resolution against energy consumption and device longevity, especially in battery-powered systems.

#### 3.1.2 Inertial measurement units (IMUs)

Working principle and measured parameters: Inertial measurement units combine triaxial accelerometers and, often, gyroscopes to capture linear acceleration and angular velocity. Numerical integration and orientation estimation algorithms can then reconstruct joint kinematics, including flexion–extension angle trajectories, step counts, cadence, and spatiotemporal gait parameters (Rattanakoch et al., 2023).

Applications and functions in smart hip and knee prostheses: Modern commercial systems, such as Persona IQ® (Rattanakoch et al., 2023; Dundon et al., 2024), embed an IMU into the tibial component to continuously track knee range of motion, activity levels, and gait quality during daily life. Unlike strain-gauge systems that focus on multi-axis load resolution in small research cohorts, IMU-centric designs emphasize scalable, long-term monitoring of functional recovery and activity phenotypes in large patient populations. These kinematic and activity metrics support orthopedic phenotyping, for example, identifying typical versus atypical recovery trajectories, “low-activity” versus “high-impact” users, or kinematic patterns suggestive of stiffness or instability.

Output signal, interfacing, and data transmission: IMUs typically provide digital outputs via Inter-Integrated Circuit (I^2^C) or Serial Peripheral Interface (SPI) interfaces, simplifying integration with on-implant microcontrollers. Sampling rates in smart knee implants are commonly in the tens of hertz range for long-term monitoring, with higher rates reserved for short diagnostic intervals if needed. Data are buffered and transmitted intermittently over low-power radio frequency (RF) links or Bluetooth-class radios to in-home receivers and cloud platforms.

Power requirements and system-level implications: Microelectromechanical systems (MEMS) accelerometers and gyroscopes can operate at microamp-level currents when duty-cycled, yielding average power consumption in the low-to mid-microwatt range at clinically relevant sampling rates. This relatively low energy cost, combined with high information yield for activity and kinematic phenotyping, is a key reason why IMU-centric architectures have become the preferred sensing core for first-generation commercial smart knees. Nevertheless, continuous operation of gyroscopes is more power-hungry than accelerometer-only modes, motivating adaptive duty-cycling and mode switching to balance kinematic fidelity with battery life.

#### 3.1.3 Thermal and environmental sensors

Working principle and measured parameters: Thermal sensors such as thermistors or resistance temperature detectors (RTDs) rely on the temperature dependence of a material’s resistance, whereas thermocouple-based sensors generate a voltage proportional to the temperature difference between junctions (Seebeck effect) (Ramanathan and Danielsson, 2001). These devices detect small changes in peri-implant temperature, typically within a physiological range of ≈20 °C–80 °C, with sub-degree resolution.

Applications and function in smart hip and knee prostheses: Telemetric hip systems have used embedded thermistors at the implant–bone or implant–bearing interface to study frictional heating during activity and to explore temperature elevations as potential early indicators of inflammation or infection (Bergmann et al., 2012; Bergmann et al., 2001). In principle, integrating thermal sensors into smart hip and knee prostheses could contribute to multimodal infection surveillance when combined with mechanical and biochemical signals.

Output signal, interfacing, and data transmission: Thermal sensors usually output analog resistance or voltage signals, read via bridge circuits or simple voltage dividers, and digitized by Analog-to-Digital Converters (ADCs). The resulting time-stamped temperature traces are transmitted using the same wireless infrastructure as mechanical and inertial data.

Power requirements and system-level implications: Given that physiological temperature shifts occur over timescales of minutes to hours rather than the millisecond dynamics of mechanical loading, thermal sensors permit low-frequency sampling (e.g., 0.1–1 Hz) and aggressive duty cycling, resulting in negligible power consumption. Consequently, they serve as advantageous auxiliary modalities in multimodal implants, enhancing diagnostic specificity through data fusion while imposing a minimal energetic penalty on the system.

#### 3.1.4 Electrochemical and biochemical sensors

Working principle and measured parameters: Electrochemical sensors transduce local chemical concentrations into electrical signals. Mechanistically, this category includes:

Conductometric sensors, which modulate resistance or impedance in response to ionic strength.

Amperometric sensors, which measure current proportional to the redox activity of analytes.

Potentiometric sensors, which detect electrical potentials driven by ion-selective interactions.

Biochemical sensors extend these principles by integrating biological recognition elements—such as enzymes, antibodies, or nucleic acid probes—to selectively target specific biomarkers (e.g., interleukin-6 (IL-6), C-reactive protein (CRP), or pathogen-specific DNA).

Applications and function in smart hip and knee prostheses: To address infection surveillance, pH-responsive hydrogels have been integrated into hip implants to monitor synovial pH shifts indicative of septic arthritis (Wijayaratna et al., 2021). Beyond pH, emerging research focuses on sensing inflammatory cytokines, specific biomarkers, and wear debris to distinguish septic from aseptic etiologies of implant failure. While promising for capturing early biological signatures of adverse tissue reactions, these technologies remain largely preclinical and await validation beyond *in vitro* or animal studies (Gray et al., 2018).

Output signal, interfacing, and data transmission: Electrochemical sensors typically generate analog currents or voltages that require specialized analog front ends (AFEs), such as low-noise transimpedance amplifiers, potentiostats, or impedance-measurement interfaces on the implant. These circuits condition and digitize slowly varying biochemical signals at low sampling rates, allowing the resulting data to be multiplexed with mechanical and inertial streams for wireless telemetry.

Power requirements and system-level implications: In contrast to resistive-load bridges or inertial-sensor front ends, electrochemical front ends tend to incur higher power demands due to the need for continuous bias currents and stable reference potentials under potentiostatic operation. However, the slow temporal dynamics of biochemical parameters permit aggressive duty cycling (e.g., infrequent “chemical snapshots”), effectively constraining average power consumption to the microwatt range. The most formidable challenge remains preserving sensor specificity and calibration stability over years in a biofouling-prone, protein-rich *in vivo* environment, while still respecting the strict volume and energy constraints of hip and knee prostheses.

#### 3.1.5 Vibration-, piezoelectric-, and inductive-based sensors

Working principle and measured parameters: Piezoelectric sensors operate via the direct piezoelectric effect, generating an electrical charge or potential in response to mechanical deformation and, conversely, deforming when an electric field is applied (reverse piezoelectric effect). Embedded piezoelectric transducers can thus capture dynamic strain and high-frequency vibration signatures of the implant–bone construct. Inductive sensors, conversely, utilize changes in coil geometry or mutual inductance, typically within passive inductor–capacitor (LC) resonant circuits, to transduce displacement or strain into resonant frequency shifts that can be interrogated wirelessly by an external reader.

Applications and function in smart hip and knee prostheses: Prototype hip implants have leveraged accelerometer-based vibration analysis to detect shifts in resonance behavior indicative of aseptic loosening, offering a postoperative, noninvasive diagnostic modality for fixation failure (Sauer et al., 2013). Similarly, inductive strain sensors integrated into experimental bone–implant constructs have enabled remote monitoring of interface micromotion via resonant-frequency shifts. Furthermore, piezoelectric elements have been investigated for their dual utility in load sensing and auxiliary energy harvesting, bridging the gap between sensing and self-powered operation (Sauer et al., 2013; Silva et al., 2013).

Output signal, interfacing, and data transmission: Piezoelectric transducers generate high-impedance charge signals or voltage transients that require specialized signal conditioning, typically charge amplifiers or high-impedance buffers, and are then digitized at sampling rates sufficient to resolve the relevant vibration spectra. In contrast, inductive sensors embedded in passive LC resonant circuits are interrogated wirelessly by external readers that detect shifts in resonant frequency, enabling battery-free, on-demand readout via inductive coupling.

Power requirements and system-level implications: Piezoelectric elements possess the unique ability to serve as both sensors and energy harvesters. Although the power harvested under realistic joint-loading conditions is currently in the microwatt range, and therefore serves primarily as a supplement to batteries, this dual capability is an important building block for the hybrid power architectures discussed in the subsequent section on power supply and energy management.

Looking ahead, emerging designs aim to integrate mechanical, thermal, and biochemical sensing within a single implant platform, enabling multimodal monitoring of implant health (Yogev et al., 2023; Rich et al., 2024).

### 3.2 Data communication

Once acquired *in vivo*, sensor data must be transmitted outside the body for storage, visualization, and clinical interpretation. As summarized in Table 3, data communication strategies for smart hip and knee implants can be broadly grouped into three main categories: (i) near-field inductive telemetry, (ii) active low-power radiofrequency (RF) links, and (iii) passive near-field devices, such as near-field communication (NFC), radio-frequency identification (RFID), or inductor–capacitor (LC) resonant tags, for on-demand data retrieval, each with distinct trade-offs in range, hardware complexity, and on-implant power consumption.

**TABLE 3:** Comparative summary of data communication strategies for smart joint prostheses.

Communication strategy	Operating principle/Range	Power consumption profile	Advantages	Limitations	Representative implementations
Near-field inductive telemetry	Magnetic coupling between coils; range: <10 cm	Active: mW range (powered externally) Standby: 0 µW (passive)	Battery-free implant; suitable for long-term raw data streaming; mature analog front-ends	Requires bulky external coil and precise alignment; sensitive to metal shielding; not suitable for continuous background monitoring	D’Lima tibial tray; Bergmann telemetric hip
Active Low-power RF (proprietary)	Custom RF transceiver (e.g., 400 MHz/2.4 GHz); range: 2–5 m	Avg: 50–200 µW Peak: 5–20 mW	Optimized protocols for specific data types; can be tailored for low latency	Non-standard hardware (needs a custom receiver); harder to integrate with smartphones/cloud	Early research prototypes (e.g., MicroStrain links)
Bluetooth® Low Energy (BLE)	Standardized 2.4 GHz digital link; range: 2–10 m	Avg: 10–100 µW (duty-cycled) Peak (Tx): ∼5–15 mW	Automated background sync; direct smartphone/cloud integration; huge commercial ecosystem	Requires an implanted battery; signal attenuation by body/metal; high peak current requires capacitors	Persona IQ® smart knee
Passive LC resonant	Analog resonance frequency shift; range: mm–cm	Implant: 0 µW (passive) Reader: Watts range	Extremely simple hardware (coil + capacitor); great for sensing strain/loosening without ICs	Analog only (no digital ID); very sensitive to distance/alignment; “reader” is bulky	Experimental loosening sensors (e.g., ancillary components)
NFC/RFID tags	Near-field digital interrogation (13.56 MHz); range: <5 cm	Implant: 0 µW (dormant) Harvested: ∼1–10 mW	Standardized digital ID; battery-free; allows “point-of-care” snapshot data readout	Intermittent only (cannot track activity history); very short range; requires reader proximity	Infection tags; implant ID tags

Abbreviations: Centimeter (cm); milliwatt (mW); microwatt (µW); radio frequency (RF); megahertz (MHz); gigahertz (GHz); meter (m); average (Avg); peak (Peak); Bluetooth Low Energy (BLE); transmission (Tx); inductor–capacitor (LC); millimeter (mm); integrated circuits (ICs); identification (ID); near-field communication (NFC); radio-frequency identification (RFID).

#### 3.2.1 Near-field inductive telemetry

Early generations of smart hip and knee implants relied heavily on near-field inductive telemetry. In these systems, an implanted coil antenna is magnetically coupled to an external coil, typically worn by the patient (e.g., in a belt or garment), which provides power and a bidirectional data link (Heinlein et al., 2009; Bergmann et al., 2012). Classic examples include D’Lima’s instrumented tibial tray and Bergmann’s telemetric hip stems, in which the external peri-knee or peri-hip coil energizes the on-implant electronics and receives multi-channel RF load signals in real time (D'Lima et al., 2007; Heinlein et al., 2009; Meswania et al., 2008; Bergmann et al., 2012). This architecture enables battery-free implants suitable for long-term implantation, with mature analog and RF front-ends and the ability to stream high-bandwidth data whenever the coupling coils are aligned. However, the effective range is short (typically <10 cm), performance is susceptible to coil alignment and metal shielding, and early belt-worn hardware was bulky. Even with later miniaturization of external coils, the need for deliberate coil positioning means that continuous, unrestricted background monitoring during everyday life is not practically achievable (Bergmann et al., 2012).

#### 3.2.2 Active low-power RF (e.g., Bluetooth® LE)

More recent, commercially deployed systems, exemplified by the Persona IQ® smart knee, integrate active, low-power RF radios within the implant to support automated, home-based data synchronization. In this architecture, the tibial component houses a low-power wireless module (e.g., Bluetooth® Low Energy) that periodically transmits kinematic and activity data to a small in-home receiver or compatible hub; the data are then securely forwarded to cloud-based platforms accessible for clinician review and analytics (Zimmerbiomet, 2025a; Zimmerbiomet, 2025b). This approach eliminates the need for wearable coils, supports near-daily or scheduled background synchronization with minimal patient interaction, and provides communication ranges of several meters within the home environment. The trade-off is that an implanted primary battery (or hybrid harvesting scheme) is required, RF antennas must be integrated into metal-constrained arthroplasty geometries, and radio operation becomes a major contributor to the overall power budget. In practice, aggressive duty-cycling keeps the average on-implant power in the low-to mid-microwatt range. Still, instantaneous transmit events reach the milliwatt level, necessitating careful power management and protocol design.

#### 3.2.3 Passive NFC/RFID/LC tags

A complementary class of communication strategies uses passive near-field devices, such as NFC, RFID, or LC resonant tags, for on-demand data retrieval (Khan et al., 2020). In these systems, the implant-side circuitry is entirely passive: small coils and capacitors form resonant circuits or backscatter tags that are interrogated by an external reader placed in close proximity during clinic visits (Khan et al., 2020; Zimmerbiomet, 2021; Singer and Robinson, 2021). Energy for readout is supplied entirely by the external reader via inductive coupling, and the implanted tag does not require a battery or active RF transmitter. This makes passive tags attractive for long-term, battery-free identification or simple diagnostic functions, with minimal on-implant component count and no continuous RF emissions. However, the readout range is very short (typically a few centimeters), interrogation requires deliberate positioning of a handheld reader over the implant, and only intermittent, point-of-care data snapshots can be obtained. As a result, passive NFC/RFID/LC approaches are not suited for continuous monitoring of activity or recovery trajectories.

For proactive, data-driven postoperative care, continuous or near-daily monitoring is generally preferable to purely episodic assessments. Near-field inductive links and passive NFC/RFID/LC tags, therefore, remain valuable for battery-free, on-demand access in research and selected clinical scenarios, whereas embedded low-power RF radios paired with home receivers currently offer the most practical path to routine, background synchronization in smart hip and knee implants (D'Lima et al., 2007; Heinlein et al., 2009; Meswania et al., 2008; Bergmann et al., 2012; Zimmerbiomet, 2025a; Zimmerbiomet, 2025b; Khan et al., 2020; Zimmerbiomet, 2021; Singer and Robinson, 2021). A comparative summary of these communication strategies, including typical ranges, bandwidth, power-consumption profiles, and representative implementations, is provided in Table 3.

### 3.3 Power supply and management

**FIGURE 4:**
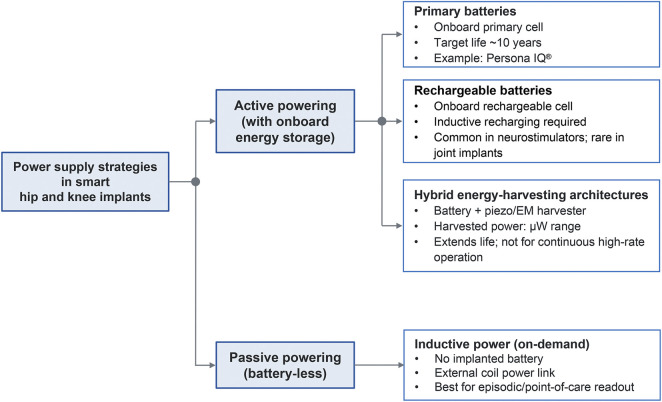
Taxonomy of power-supply strategies for smart hip and knee prostheses.

Powering implantable sensors represents a critical technical challenge. Currently, the available solutions can be categorized into active (with onboard energy storage) and passive (battery-less) power supplies (Figure 4).

#### 3.3.1 Active powering: primary battery-powered

Battery-powered implants provide autonomy and enable continuous sensing without external alignment. The Persona IQ® smart knee adopts this approach, using a medical-grade primary battery with a service life of at least 10 years (Zimmerbiomet, 2021). To conserve energy, devices typically collect data continuously but transmit it intermittently via aggressive duty cycling. Key design priorities include hermetic sealing to prevent leakage, rigorous lifetime energy budgeting to cover the expected implant horizon (often 15–20 years), and graceful degradation protocols: when end-of-life is reached, sensing functions cease, but the prosthesis must remain mechanically safe (Singer and Robinson, 2021). Advances in battery chemistry and ultra-low-leakage microelectronics continue to extend effective service lifetimes (Sheng et al., 2021).

#### 3.3.2 Active powering: wireless recharging

Rechargeable cells can be replenished wirelessly (e.g., via inductive links) to avoid full dependence on a finite primary battery. This approach theoretically extends device longevity but shifts the engineering burden to charge management, thermal limits (to prevent tissue heating), and coil alignment tolerance. While commonplace in neurostimulators and some cardiac devices, this modality is less common in current orthopedic sensors because of the added hardware complexity and the burden on patients to perform regular charging sessions.

#### 3.3.3 Active powering: energy harvesting

Energy harvesting from patient movement or body heat is attractive primarily as a supplement to batteries (hybrid designs) rather than as a stand-alone source for continuous operation. Piezoelectric or electromagnetic harvesters convert kinetic energy from gait cycles into electrical energy, which is buffered in capacitors or rechargeable batteries (Kim et al., 2023). For example, Silva et al. proposed a hip implant that integrates multiple harvesters to capture mechanical energy during walking (Silva et al., 2013). However, under realistic joint-loading conditions, the available power is typically in the microwatt range, orders of magnitude below the requirements for high-bandwidth sensing and RF transmission. Consequently, the realistic near-term role of energy harvesting is to extend battery life and reduce the frequency of battery-dependent events, particularly in active patients. Truly battery-free operation via harvesting is likely limited to low-duty-cycle, intermittent transmissions once sufficient charge has accumulated (Safaei et al., 2018). Thermal approaches, while conceptually interesting, typically yield insufficient power at this anatomical site to be viable.

#### 3.3.4 Passive powering: inductive power

Early smart implants relied on inductive coupling for wireless power from the outside, similar to RFID tags. These implants contain no battery but receive power on demand from an external electromagnetic source (Covaci and Gontean, 2020). While this architecture obviates battery longevity constraints and, in principle, enables indefinite operation, it requires an external reader coil in close proximity (Hannan et al., 2014). This requirement limits the system to episodic, point-of-care data retrieval, potentially missing transient events, as continuous monitoring would require the patient to wear bulky external hardware for prolonged periods (Hannan et al., 2014).

Regardless of the power strategy, all on-implant electronics must be meticulously engineered for minimal power consumption. The use of ultra-low-power microcontrollers, optimized sensor duty cycling, and energy-efficient telemetry protocols has been critical to sustaining durable *in vivo* operation.

### 3.4 Data processing and storage

Given that implant size, power budget, and onboard computing capabilities are tightly constrained, current smart implants typically transmit only raw or minimally preprocessed data to an external receiver for back-end processing. On the implant side, computationally lightweight preprocessing, including basic filtering, decimation, timestamping, and light compression, is executed on the implant’s microprocessor before transmission (Narasimhan et al., 2010), whereas substantial processing and clinical interpretation occur off-implant.

To ensure continuity of data despite intermittent connectivity, smart implants incorporate onboard nonvolatile memory to support a store-and-forward workflow. A typical design utilizes a small ring buffer to cache time-stamped sensor streams for a defined retention window; once connectivity is restored, the buffered data are uploaded in order, and the memory is freed for new recordings. For example, the Persona IQ® continuously records sensor data and uploads it to the home base station when in range, relying on built-in temporary storage for link outages (Zimmerbiomet, 2025a). Practical design considerations for these storage subsystems include buffer depth, flash memory write-endurance, data integrity checks, and on-device encryption. Looking ahead, the integration of advanced on-implant processing (“edge computing”) is expected to enable real-time event detection, pattern recognition, and selective transmission of clinically salient episodes or alerts. While increasing onboard computational load typically raises power consumption, edge computing can, in principle, yield net energy savings by drastically reducing the radio duty cycle, transmitting only key alerts rather than continuous raw streams. In practice, achieving this efficiency requires ultra-low-power processor architectures and sophisticated, event-driven algorithms that triage continuous telemetry into compact features and alerts suitable for downstream analysis driven by Artificial Intelligence (AI) and Clinical Decision Support Systems (CDSS). Under these conditions, edge capabilities can expand diagnostic functionality while maintaining or even lowering the total energy cost per unit of clinical insight, thereby enhancing both implant longevity and operational efficiency.

### 3.5 Biocompatibility and device encapsulation

The integration of electronic components into joint implants raises critical biocompatibility and safety considerations. Sensors, circuits, antennas, and power sources must be hermetically sealed from body fluids to prevent corrosion and the release of harmful leachables, with materials and encapsulation stacks selected and tested in accordance with ISO 10993 and related implantable-device standards (Lu et al., 2023). To ensure long-term reliability, a hierarchical packaging strategy is often employed: active electronics are typically housed within hermetically sealed titanium enclosures or protected by glass–metal feedthroughs that form a long-term diffusion barrier against moisture ingress and ion transport. Conversely, passive components or flexible interconnects may rely on biocompatible polymeric coatings—such as parylene-C, medical-grade silicone, epoxy encapsulants, or polyimide, to provide electrical insulation and secondary chemical protection (Winkler et al., 2017).

Structurally, smart implants continue to utilize established orthopedic biomaterials, including Ti-6Al-4V and CoCrMo alloys for load-bearing components and ultra-high-molecular-weight polyethylene (UHMWPE) for bearing surfaces; in some designs, ceramic heads (e.g., alumina or zirconia) and porous tantalum structures are also employed to optimize wear and osseointegration. However, the integration of sensor modules requires meticulous mechanical engineering to maintain the implant’s structural integrity. Internal cavities or embedded modules within load-bearing regions can act as stress raisers, potentially compromising fatigue strength. Accordingly, electronic modules are preferentially positioned in mechanically shielded zones, such as the hollow interior of stem extensions in knee implants or near the neutral axis of the femoral neck region in hip prostheses, to minimize disruption of established load paths and ensure longevity of the prosthesis (Bergmann et al., 2012; Silva et al., 2013). These modified geometries must subsequently undergo fatigue and wear testing to the same or higher standards as conventional implants to verify that encapsulation features do not introduce new failure modes.

### 3.6 Imaging and interference

Compatibility with diagnostic imaging modalities, particularly magnetic resonance imaging (MRI) and computed tomography (CT), constitutes a critical safety and functional requirement (FDA, 2023). While conventional metallic implants generate passive susceptibility artifacts, the integration of active electronics introduces additional risks, including radiofrequency-induced heating, magnetically induced displacement or torque, and potential device malfunction (e.g., unintended resets or induced currents) during MRI examinations (Al-Dayeh et al., 2020). Furthermore, high-density electronic components, such as batteries and shielding cans, can exacerbate beam-hardening artifacts in CT, potentially obscuring the bone–implant interface and complicating postoperative assessment. Consequently, smart implants are almost exclusively labeled as “MR conditional” rather than “MR safe”. Regulatory approval necessitates rigorous testing to define safe operating windows, including limits on static field strength (e.g., 1.5 T or 3 T), spatial gradient fields, and specific absorption rate (SAR) (FDA, 2023; Kuroda and Yatsushiro, 2022). Manufacturers must provide precise labeling and clear guidance for radiology teams, as failure to adhere to these parameters could result in patient injury or device malfunction.

In summary, contemporary smart joint prostheses exemplify the convergence of multidisciplinary technologies, including microscale sensors, ultra-low-power integrated circuits, short-range wireless links, and hermetic biocompatible packaging. Advances in MEMS technology have been pivotal, enabling miniaturized, energy-efficient sensing and limited on-implant processing units to be seamlessly incorporated into implants without compromising implant geometry or mechanical performance (Chircov and Grumezescu, 2022). Ultimately, successful smart implant design requires holistic optimization: maximizing sensing fidelity and telemetry performance while strictly adhering to the constraints of structural integrity, imaging compatibility, and long-term biocompatibility.

## 4 Clinical applications and early outcomes of smart prostheses

After years of laboratory development, sensor-integrated prostheses are beginning to transition into clinical practice. The initial clinical implementations of smart implants have provided valuable real-world data on joint function outside clinical settings, highlighting the potential benefits of continuous monitoring to augment postoperative care, while also underscoring the limitations of the current evidence base. This section summarizes key clinical studies and practical applications of fully implantable smart knee and hip prostheses, together with early insights and remaining evidence gaps (Table 4).

**TABLE 4:** Comprehensive summary of key clinical and preclinical studies on sensor-integrated hip and knee prostheses.

Study/system	Implant type	Sensing technologies	Measured parameters	Cohort and monitoring duration	Communication and power	Key findings	Key limitations
Taylor et al. (1998)	Knee; instrumented hinged distal femoral prosthesis (tumor resection/DFR)	Strain gauges; inductive telemetry	Joint force (telemetric load signal during gait/functional tasks)	Clinical proof-of-concept; N = 1 (reported); session-based measurements	Near-field inductive telemetry; externally powered (coil); real-time when coupled	• First patient-derived telemetric force data reported from a knee implant • Demonstrated the feasibility of fully implantable wireless joint-force monitoring	• Single-patient, specialized/custom implant (not standard primary TKA) • External coil alignment and dedicated hardware; limited free-living monitoring
D’Lima instrumented tibial tray (2004–2005)	Knee; TKA instrumented tibial tray	4 uniaxial load cells/strain-gauge bridges	Axial tibial forces; medial–lateral load distribution; center of pressure (surrogate)	Clinical research; N = 1–3 (reported); short- to mid-term monitoring (lab + ADLs)	Inductive coupling + RF telemetry; externally powered (coil)	• Peak loads ∼2.5–3× BW during level walking; higher during stairs/sit-to-stand • Provided early *in vivo* tibial force datasets for TKA biomechanics	• Very small cohorts; primarily research endpoints • Limited to axial compression in early versions; external coil/user burden
Meswania et al. (2008)	Knee; instrumented knee prosthesis (long-stem/tumor or revision-type design)	Embedded sensors (likely strain-based); on-implant telemetry (platform-specific)	*In vivo* load/strain metrics (details platform-dependent/reported in original study)	Clinical/experimental platform; cohort size and follow-up not consistently reported across prototypes	Telemetric readout (details not reported here); likely inductive/near-field coupling in early designs	• Contributed to early generations of instrumented knee implants for *in vivo* biomechanics	• Highly specialized implant and limited availability • Insufficient publicly reported cohort size/long-term monitoring; limited generalizability
Heinlein et al. (2007), Heinlein et al. (2009)	Knee; instrumented tibial component (INNEX™ fix knee platform)	12 strain gauges (multi-bridge); 6-DOF force–moment reconstruction	3D forces (fx, fy, fz) + 3 moments (Mx, My, Mz); compartmental load patterns during ADLs	Clinical research; n = 5 (reported) with mid-term follow-up; session-based ADL testing	Inductive coupling + RF telemetry; externally powered; real-time when coupled	• Peak tibiofemoral forces ∼3–4× BW in routine activities; higher during stairs • Task-dependent variations in compartmental load sharing; high-value reference datasets	• Complex manufacturing and calibration; potential *in vivo* drift/long-term signal stability issues • External hardware and short-range coupling; small cohorts; not scalable to routine care
Crescini et al. (2011)	Knee; instrumented TKA insert (polyethylene)/sensing module	Magnetoresistors + magnets; near-field antenna (∼125 kHz)	Magnetic-field–based kinematic/contact surrogates (e.g., relative position/contact point proxies)	Preclinical/Benchtop validation (not applicable)	Near-field inductive/NFC-like readout (125 kHz); externally powered during interrogation	• Demonstrated the feasibility of embedding magnetic sensing elements within an arthroplasty construct	• Preclinical stage; limited *in vivo* validation and long-term durability data • Signal sensitivity to alignment, metal environment, and encapsulation constraints
Safaei et al. (2020)	Knee; TKA insert concept	Piezoelectric (PZT) transducers integrated into the insert	Load/pressure-related signals; potential auxiliary energy harvesting (conceptual)	Preclinical/Benchtop validation (not applicable)	Not consistently reported; typically requires front-end conditioning; harvesting is viewed as supplemental	• Proposed piezoelectric insert architecture for force/pressure sensing in TKA • Highlighted potential for combining sensing with limited energy harvesting	• Primarily preclinical; packaging, stability, and calibration remain challenging • Harvestable power under realistic conditions is typically microwatt-level (supplemental only)
Persona IQ® smart knee (2021)	Knee; commercial TKA tibial stem extension module	IMU (triaxial accelerometer + gyroscope) ± auxiliary sensors (e.g., temperature)	ROM trajectories; step count/cadence; walking speed/activity metrics; kinematic phenotypes	Post-market observational cohorts; n > 200 reported; longitudinal home monitoring	Primary battery (reported service life up to ∼10 years); low-power RF/Bluetooth® LE-class link to home hub → cloud	• Enables remote recovery surveillance and longitudinal functional monitoring • Provides objective kinematic/activity data complementary to PROMs	• Does not directly measure joint loads/forces or ligament tensions • Clinical outcome impact and cost-effectiveness still under evaluation; requires home hub/adherence and CDSS/workflow integration
Bergmann telemetric hip/OrthoLoad platform (1984–2012)	Hip; THA instrumented femoral stem/neck (telemetric)	Strain gauges (3D loads ± moments); thermistors in some versions (temperature)	3D hip contact forces across ADLs; interface temperature profiles (selected systems)	Small, highly selected cohorts; repeated session-based measurements; long-term datasets archived (OrthoLoad)	Inductive coupling + RF telemetry; externally powered (coil); battery-free when coupled	• Established benchmark *in vivo* hip loading datasets (walking commonly ∼2–3× BW; higher in demanding tasks) • OrthoLoad enabled broad reuse for model validation, simulator design, and implant benchmarking	• Research-grade platform; limited sample sizes and generalizability • Requires external coil/alignment; potential drift and long-term stability considerations
Silva et al. (2013)	Hip; THA prototype with integrated harvesters	Electromagnetic + piezoelectric generators; power management electronics (prototype)	Harvested energy/power from gait; feasibility of hybrid powering for implant electronics	Preclinical/Benchtop validation (not applicable)	Hybrid powering (harvesting → energy reservoir); prototype control/antenna modules (e.g., BLE-class components shown)	• Demonstrated the feasibility of integrating multiple harvesters and power management within a hip construct • Motivated hybrid architectures to extend battery life rather than enable fully battery-free operation	• Harvested power is typically microwatt-range under realistic joint loading; insufficient for continuous sensing + RF transmission • Added mechanical/electrical complexity; not yet clinically validated
Wijayaratna et al. (2021)	Hip; prototype sensorized THA component (biochemical/electrochemical concept)	Hydrogel + radiopaque marker + radiographic readout	Local biochemical marker(s) (e.g., pH or infection-related signals; study-specific)	Preclinical/Benchtop validation (not applicable)	Interrogation/power strategy study-specific (often on-demand readout in early prototypes)	• Illustrates the feasibility of integrating biochemical sensing elements into hip prosthesis constructs for infection surveillance	• Preclinical stage; specificity, biofouling, and calibration drift remain major barriers • Long-term stability and clinically validated outcome linkage not yet established

Abbreviations: total hip arthroplasty (THA); total knee arthroplasty (TKA); total joint arthroplasty (TJA); degrees of freedom (DOF); six degrees of freedom (6-DOF); range of motion (ROM); activities of daily living (ADL[s]); body weight (BW); hertz (Hz); radio frequency (RF); near-field communication (NFC); radio-frequency identification (RFID); inductor–capacitor (LC); Bluetooth Low Energy (BLE/LE); inertial measurement unit (IMU); patient-reported outcome measures (PROMs); sample size (n); U.S., Food and Drug Administration (FDA).

In TKA, early instrumented implants developed in the 2000s were initially tested in small patient cohorts as part of clinical research (D'Lima et al., 2005; D'Lima et al., 2006; D'Lima et al., 2007). D’Lima et al. implanted their first-generation load-sensing tibial component in a patient and successfully recorded *in vivo* tibial forces during daily activities such as walking, chair rising, and stair climbing (D'Lima et al., 2005; D'Lima et al., 2006; D'Lima et al., 2007). They reported that the peak loads were approximately 2.5–3 times the body weight during level walking, with even higher loads during more strenuous activities (D'Lima et al., 2007). Subsequent studies using advanced six-axis sensor implants monitored multiple patients over extended periods. Kutzner et al. measured knee joint loads in five TKA recipients performing activities of daily living and reported peak contact forces of 3.3 times the body weight during level walking, with even greater loads observed during stair ascent and descent (Kutzner et al., 2010). Their data further revealed variations in medial and lateral compartment load sharing during dynamic daily activities (Kutzner et al., 2010), information that previously was available only from computational models.

Clinically, these measurements have informed patient counseling on the risks of high-impact activities and have shaped postoperative rehabilitation protocols to minimize excessive joint loads during early recovery (Kutzner et al., 2010). The datasets also highlight substantial interpatient variability: some individuals consistently generate higher loads during identical activities, which could help explain the difference in implant longevity. In turn, objective load metrics have the potential to support more personalized recommendations for activity levels and rehabilitation strategies. However, these links remain largely hypothesis-generating; causal relationships between load-guided recommendations and improved long-term survivorship or function have not yet been demonstrated in prospective, outcome-powered studies (Damm et al., 2019; Haffer et al., 2022).

In THA, instrumented implants have similarly advanced our understanding of joint mechanics. Since the 1990s, Bergmann et al. have implanted telemetered hip prostheses and earlier instrumented femoral implants, enabling direct measurement of hip joint contact forces across daily activities such as walking and cycling (Bergmann et al., 1993). The publicly accessible OrthoLoad database comprehensively documents these *in vivo* load measurements (Orthoload, 2025; Aschenbrenner et al., 2025; Bergmann et al., 1994; Bergmann et al., 1995; Bergmann et al., 1997). These studies revealed that routine activities, such as normal walking, frequently generate hip contact forces of 2–3 times body weight, with higher forces during more strenuous tasks, such as stair climbing or load carrying (Orthoload, 2025; Aschenbrenner et al., 2025; Bergmann et al., 1994; Bergmann et al., 1995; Bergmann et al., 1997). These biomechanical insights have informed safer rehabilitation protocols and guided improvements in implant design to better withstand realistic *in vivo* loads. Furthermore, the addition of temperature sensors provided novel *in vivo* data on implant interface temperatures (Bergmann et al., 2012). In well-functioning implants, interface temperatures remain stable during routine activities, whereas atypical elevations may indicate complications such as periprosthetic infection or excessive friction resulting from component malposition (Bergmann et al., 2012; Bergmann et al., 2001). These observations support continuous temperature monitoring as a promising early warning metric, although validation in larger cohorts with outcome-linked endpoints is still required.

While these early studies focused primarily on biomechanical validation and mechanistic understanding, the overarching goal is to improve patient outcomes. The advent of the first FDA-approved, commercially available smart knee, Persona IQ®, has enabled broader evaluation of clinical utility in routine practice. The device functions as a conventional TKA while continuously recording knee kinematics and activity for remote review. Early clinical experiences have suggested potential benefits in several domains:

- Remote recovery surveillance: device-derived kinematics can flag suboptimal progress earlier than routine visits. For instance, in a series of 182 TKA recipients, two patients with persistently limited flexion were identified early and underwent earlier-than-usual manipulation under anesthesia to address arthrofibrosis (Yocum et al., 2023). However, whether such earlier interventions translate into durable improvements in long-term function or survivorship, or merely increase the intensity and cost of postoperative care, remains uncertain.
- Complementary assessment: a comparative study revealed that sensor-derived gait metrics and patient-reported outcome measures (PROMs) both improved postoperatively but correlated weakly following TKA (Guild et al., 2024), suggesting complementary roles of implant metrics and PROMs for more comprehensive postoperative evaluation (Guild et al., 2024). This underscores that implant-derived telemetry and PROMs capture partly orthogonal aspects of recovery: objective, continuous performance on the one hand, and patient-perceived pain and function on the other. As such, they should be viewed as complementary, rather than competing, endpoints within a comprehensive postoperative evaluation framework (Guild et al., 2024).
- Reducing the burden of follow-up: when home-synchronized data are reassuring, some routine in-person visits can be replaced by telemedicine without compromising care (Orthofeed, 2024). This may improve convenience and clinical efficiency, and could increase follow-up adherence, thereby indirectly supporting long-term clinical outcomes, although this hypothesis requires confirmation in broader health-economic and outcome studies.

Despite these promising observations, the adoption of smart prostheses remains at an early stage. The Persona IQ® is currently the only FDA-approved smart joint implant, and its use remains relatively limited as healthcare providers continue to evaluate its practical utility. To date, most clinical evidence derives from observational cohorts with short-to mid-term follow-up and without randomized comparisons against standard care. Definitive evidence of improved long-term survivorship, lower revision rates, or consistently superior PROMs is not yet available. Additionally, critics note that some clinical targets, such as malalignment and periprosthetic infection, can often be monitored by less invasive means (e.g., periodic imaging and laboratory testing), albeit less continuously and less comprehensively.

Another concern is whether the “data deluge” from continuous monitoring genuinely enhances clinical decision-making. As one commentator remarked, “If a knee talks, who’s listening?” (Medtechdive, 2022). To make these data usable at scale, smart implant programs will require true clinical decision-support systems (CDSS), not merely raw dashboards that triage continuous data streams into prioritized alerts, risk scores, and interpretable trend summaries integrated into routine workflows. Without such tools, there is a real risk that additional data could exacerbate clinicians’ cognitive load rather than improve decision-making. Conversely, well-designed CDSS could help shorten the learning curve, standardize responses to telemetry-derived findings, and ensure that attention is directed to those patients most likely to benefit from timely intervention.

Looking forward, fully implantable smart hips are anticipated in the near future. Although no commercial device is yet available, companies such as Canary Medical have announced plans to adapt their sensor technologies for THA and other orthopedic implants (Orthostreams, 2021). As evidence accumulates, the clinical role of smart prostheses will ultimately be defined less by technical novelty than by demonstrations of outcome impact, cost-effectiveness, and seamless integration into care pathways, including clear protocols for when and how telemetry-derived information should change management.

## 5 Challenges and considerations

Despite the growing enthusiasm for smart joint prostheses, several hurdles must be addressed before widespread clinical adoption. Key challenges encompass device miniaturization and design constraints, power supply and longevity, demonstrable clinical utility, regulatory clearance, biocompatibility and safety, data privacy, data overload, and workflow integration. This section summarizes these issues and outlines considerations for successful implementation in routine clinical practice.

### 5.1 Device miniaturization and design constraints

Standard joint implants provide negligible spare volume for integrating auxiliary electronics. Incorporating sensors, circuitry, and batteries often necessitates modified geometry or stem extensions; for instance, the Persona IQ® sensor module requires a 58-mm tibial stem extension, mandating the use of a stemmed baseplate and additional bone resection compared with standard primary implants (Kelmers et al., 2022). This requirement may limit applicability in patients with smaller anatomy or compromised bone quality (Farazin and Darghiasi, 2025; Lee et al., 2025). Furthermore, introducing internal cavities or embedding sensors within implants may lead to stress concentrations, potentially compromising structural integrity and reducing fatigue strength. Ensuring that sensor-integrated implants withstand physiological loading conditions without mechanical degradation requires rigorous finite element analyses, extensive fatigue testing, and iterative design refinement. While advances in MEMS and high-density packaging can help mitigate these constraints, achieving seamless integration without biomechanical compromise remains a fundamental engineering challenge.

### 5.2 Power supply and longevity

A durable power supply remains a critical constraint for smart implants. Since surgical replacement or recharging is generally impractical, batteries define the device’s functional lifespans. While current designs target operational periods of approximately 10 years, this duration often falls short of the mechanical longevity of modern prostheses (de Almeida Fernandes et al., 2023). Once the battery depletes, the prosthesis remains mechanically functional but loses its “smart” capabilities, thereby diminishing its long-term cost-effectiveness. Although energy harvesting offers a theoretical solution, its clinical reliability remains unproven. Realistically, under typical joint-loading conditions, harvested power is in the microwatt range and is best viewed as a supplement to batteries in hybrid architectures rather than a standalone source for fully battery-free continuous operation. Additionally, safety by design is paramount; depleted batteries or failed circuits must remain hermetically sealed to prevent leakage of hazardous materials. Ideally, future electronics should either be energy-efficient enough to match the implant’s mechanical service life or support noninvasive transcutaneous recharging. Until then, tight power budgets will continue to impose strict design boundaries.

### 5.3 Data overload and clinical utility

As the volume of telemetry data increases, clinical interpretation becomes increasingly complex, as most orthopedic surgeons are not trained to analyze continuous time-series and large kinematic datasets. In practice, edge-computing architectures where initial feature extraction and event detection occur on the implant or a nearby hub, combined with AI-driven analytics for risk stratification and outcome prediction, will be essential to reduce bandwidth, lower radio duty cycles, and manage clinician cognitive load, rather than optional “nice-to-have” additions. Translating raw data into actionable information, such as establishing decision thresholds for intervention, is essential. Additionally, demonstrating tangible clinical benefits is critical for justifying the added cost of smart implants. To date, no study has conclusively shown that smart implants improve long-term survival outcomes (Gordon et al., 2025). To warrant widespread adoption, smart systems must offer distinct advantages over non-invasive alternatives (e.g., external wearables), such as measuring internal compartmental loads or detecting early signs of loosening or infection. Rigorous prospective trials are required to determine whether these capabilities truly translate into reduced complication rates and improved patient satisfaction.

### 5.4 Regulatory hurdles

Sensorized implants are regulated as active medical devices (Class II/III) and undergo more rigorous approval processes. The development timeline is lengthy: for example, Persona IQ® took approximately 7 years from the initial prototype to FDA clearance, necessitating significant investment (∼$46 million) and extensive verification (Kelmers et al., 2022). New devices, particularly those employing novel sensing modalities, face stringent scrutiny. Sponsors must demonstrate electrical safety, electromagnetic compatibility, battery reliability, cybersecurity, and mechanical integrity, while ensuring that smart components do not hinder future revision surgeries. Recent regulatory discussions, including the FDA’s “SMART” device workshops, highlight that evaluation of such implants must extend beyond hardware to encompass software validation, algorithm robustness, cybersecurity-by-design, data integrity, and robust post-market performance monitoring, reflecting an emerging regulatory-science framework tailored to sensorized orthopedic devices.

### 5.5 Biocompatibility and safety

Smart implants entail conventional surgical risks while introducing electronics-related concerns, such as potential wear debris, corrosion, or tissue reactions to sensors, antennas, and interconnects. A critical design philosophy is fail-safe operation: electrical failures must not result in overheating, unintended current leakage, or mechanical compromise. Basic redundancy should be considered to preserve data integrity in the event of partial sensor failure (Majumdar et al., 2024). Furthermore, as electronic implants, these devices require adherence to specific MRI protocols. Although few adverse events have been directly attributed to electronic components to date, ongoing vigilance and post-market surveillance remain essential as clinical adoption expands (Abyzova et al., 2023).

### 5.6 Data privacy and ethics

The continuous generation of personal health data raises critical questions regarding ownership, stewardship, and cybersecurity (Pycroft and Aziz, 2018). Manufacturers have attempted to mitigate these risks through rigorous encryption and anonymization protocols. For instance, Zimmer Biomet proactively records only essential kinematic metrics without personal identifiers or GPS/audio information (Zimmerbiomet, 2025c; Wakale and Goswami, 2025). However, ethical concerns persist regarding the secondary use of aggregated proprietary data for commercial research or without explicit patient consent. Future governance frameworks must strike a balance between clinical and scientific utility and stringent privacy protection to maintain patient privacy and autonomy (Harmon et al., 2015; Baumann et al., 2021).

In summary, smart prostheses hold substantial promise but face significant technological, clinical, regulatory, ethical, and economic challenges. Although the pathway from prototype to widespread clinical adoption is complex, precedents in other active implants (e.g., cardiac pacemakers and neurostimulators) suggest that these obstacles are surmountable. With continued interdisciplinary innovation and with careful attention to power budgets, regulatory requirements, data governance, and edge/AI-enabled decision support, orthopedic smart implants are poised to follow a similar trajectory toward mainstream clinical integration.

## 6 Future outlook

The field of fully implantable smart joint prostheses is advancing rapidly, with forthcoming innovations poised not only to mitigate current limitations but also to expand clinical capabilities in a stepwise fashion.

### 6.1 Multimodal sensor fusion

Future architectures are likely to evolve from single-domain sensing to multimodal surveillance, synergizing kinematics, internal loading, micromotion, and biochemical markers (e.g., temperature, pH, and specific enzymes). This fusion could enable cross-validated detection of infection, loosening, or imbalance earlier than any single modality could achieve (Drazan et al., 2018; Kassanos and Hourdakis, 2024; Zhang et al., 2024; Collo et al., 2014; Youssef et al., 2024; Ghanem, 2024). For instance, coupling inertial sensing for function with load sensing for mechanics can elucidate the interplay between patient activity patterns and implant biomechanical responses (Collo et al., 2014), thereby refining orthopedic phenotyping beyond purely kinematic descriptors.

### 6.2 Next-generation power and connectivity

To better match the longevity of modern implants, power strategies are targeting 15–20 years battery lifespans or efficient wireless charging capabilities (Bruschini et al., 2022). Concurrently, communication protocols may shift toward direct, secure smartphone links, potentially obviating the need for proprietary home base stations. This would facilitate near–real-time alerts and seamless integration with electronic health records (EHRs), and rehabilitation dashboards, effectively closing the loop between patient and provider. However, realizing these scenarios will require rigorous validation of long-term battery performance, wireless safety, and cybersecurity, as well as careful alignment with emerging regulatory expectations.

### 6.3 AI and predictive analytics

At the scale of population-level datasets, AI is likely to be indispensable for extracting clinically actionable patterns. Large, multipatient cohorts can define normative recovery benchmarks stratified by demographic and implant class, while predictive models can isolate early deviations linked to complications (Ghanem, 2024). For example, AI algorithms may detect subtle postoperative loading signatures that presage loosening, enabling preemptive intervention. Ultimately, coupling implant telemetry with AI-enabled Clinical Decision Support Systems (CDSS) will be essential for shifting care from reactive to proactive: identifying potential complications before they become clinically evident, while mitigating information overload at the point of care (Georgiakakis et al., 2024). Equally important will be transparent model development, prospective validation, and regulatory-grade monitoring of algorithm performance over time.

### 6.4 Expansion to new indications

Beyond hips and knees, the “smart” concept is expanding to other anatomical regions. Sensorized shoulder prostheses could monitor rotator cuff forces or detect impingement episodes (Bergmann et al., 2007; Westerhoff et al., 2009). In spinal surgery, instrumented rods or interbody devices are being designed to monitor load-sharing and the progression of spinal fusion (Canarymedical, 2024). Similarly, smart fracture fixation devices (plates and nails) could monitor fracture healing via onsite strain sensing, providing early warning of non-union or instability (Jeyaraman et al., 2023). These applications benefit from the foundational models established by smart joint prostheses, though each presents distinct biomechanical and clinical challenges that will require indication-specific solutions rather than direct transplantation of hip and knee designs.

### 6.5 Biocompatible and flexible electronics

Future hardware will move away from bulky rigid modules. Flexible electronics and conductive polymers could be embedded directly into implant surfaces or coatings, thereby minimizing mechanical compromise (Tsikriteas et al., 2020). Furthermore, advancements in bio-MEMS present opportunities for highly sensitive, cellular-level sensing, such as detecting pathogen-specific enzymes to offer earlier and more specific infection diagnosis compared to generic pH sensors (Ramesh et al., 2022). These trends indicate that increasingly sophisticated, unintrusive sensing layers are feasible in the foreseeable future (Chircov and Grumezescu, 2022), provided that long-term stability, encapsulation, and manufacturing reproducibility can be demonstrated.

### 6.6 Data-driven design and personalization

Continuous real-world feedback loops will progressively influence implant development. By revealing patient-specific loading phenotypes, smart implants enable manufacturers to optimize next-generation geometries and materials to match *in vivo* loading conditions. Ultimately, this could enable personalized smart implants: devices with customized geometry, sensor configurations, and power policies tuned to the individual’s anatomy and anticipated activity profile, thereby potentially enhancing both longevity and clinical outcomes.

### 6.7 Toward closed-loop therapeutics

The longer-term vision involves closed-loop therapeutic implants: devices that not only sense internal conditions but also respond autonomously. Potential capabilities include on-board drug delivery (e.g., releasing antibiotics/anti-inflammatory agents from integrated reservoirs when infection/inflammation signatures are detected) and micro-actuated alignment adjustments when adverse loading patterns emerge. Early analogs, such as remotely adjustable spinal rods and magnetically controlled growing rods, have demonstrated the feasibility of mechanically adaptive implants.

Coupling such actuation with internal sensing could enable semi-automated, condition-responsive care, which represents the ultimate convergence of diagnostics and therapy within a single implant. Although such fully closed-loop systems remain speculative at present, articulating this trajectory helps to position current smart implants as foundational steps on a continuum from monitoring toward intelligent, therapeutic devices.

## 7 Limitations

This review has several limitations that should be acknowledged. First, given the rapidly evolving nature of smart implant technology, some of the latest developments or unpublished innovations may not be captured. Second, the scope focuses primarily on knee and hip implants, and smart implants in other anatomical locations (e.g., shoulder, spine) are only briefly discussed and not exhaustively reviewed. Third, as a narrative rather than a systematic review, article selection and emphasis may introduce some selection bias, despite efforts to include representative and influential studies. Fourth, owing to the multidisciplinary nature of this topic, a detailed exploration of regulatory, economic, and ethical issues is necessarily selective rather than exhaustive and warrants further in-depth investigation. Additionally, while key clinical studies and technological advances were highlighted, systematic evaluation or meta-analysis extends beyond the scope of this narrative review. Finally, the real-world effectiveness and long-term outcomes of smart implants remain incompletely characterized; thus, future systematic reviews or randomized controlled trials will be necessary to definitively establish their clinical benefit and cost-effectiveness.

## 8 Conclusion

Driven by rapid advances in miniaturization, wireless telemetry, and biosensing, the outlook for fully implantable smart joint prostheses is highly promising yet still dependent on robust clinical validation. By enabling continuous, objective orthopedic phenotyping and integration with AI-enabled CDSS, these technologies are positioned to help shift orthopedic care from reactive treatment to proactive, real-time monitoring with earlier complication detection. Although challenges remain, they are being actively addressed by multidisciplinary teams. Over the next decade, smart implants are likely to progress from niche technologies to routine clinical practice, provided that robust long-term studies demonstrate clear clinical benefit and cost-effectiveness. If these conditions are met, smart prostheses may improve implant longevity and patient outcomes, enabling personalized, data-driven care that allows patients and clinicians to anticipate and mitigate complications rather than react to them.
